# Planning Abilities in Bilingual and Monolingual Children: Role of Verbal Mediation

**DOI:** 10.3389/fpsyg.2018.00323

**Published:** 2018-03-14

**Authors:** Ishanti Gangopadhyay, Margarethe McDonald, Susan Ellis Weismer, Margarita Kaushanskaya

**Affiliations:** ^1^Department of Communication Sciences and Disorders, University of Wisconsin–Madison, Madison, WI, United States; ^2^Waisman Center, University of Wisconsin–Madison, Madison, WI, United States

**Keywords:** bilingualism, planning, articulatory suppression, motor suppression, Tower of London

## Abstract

We examined the role of verbal mediation in planning performance of English–Spanish-speaking bilingual children and monolingual English-speaking children, between the ages of 9 and 12 years. To measure planning, children were administered the Tower of London (ToL) task. In a dual-task paradigm, children completed ToL problems under three conditions: with no secondary task (baseline), with articulatory suppression, and with non-verbal motor suppression. Analyses revealed generally shorter planning times for bilinguals than monolinguals but both groups performed similarly on number of moves and execution times. Additionally, bilingual children were more efficient at planning throughout the duration of the task while monolingual children showed significant gains with more practice. Children’s planning times under articulatory suppression were significantly shorter than under motor suppression as well as the baseline condition, and there was no difference in planning times between monolingual and bilingual children during articulatory suppression. These results demonstrate that bilingualism influences performance on a complex EF measure like planning, and that these effects are not related to verbal mediation.

## Introduction

Planning is a complex executive function (EF) task that entails evaluation and selection of an appropriate sequence of behaviors that will lead to the desired goal. Planning skills have been tightly linked with academic achievement in children during elementary and middle school years (Bull et al., 2008; Clark et al., 2010; Friedman et al., 2014). While a number of studies have examined planning abilities in monolingual children (Fernyhough and Fradley, 2005; Lidstone et al., 2010, 2012), only one study has examined planning in bilingual children (Jalali-Moghadam and Kormi-Nouri, 2015). Planning is a complex EF task that likely implicates multiple simple EF skills like inhibitory control, updating, and switching (Miyake and Friedman, 2012). Since bilingualism has been associated with higher performance on simple EF tasks like inhibitory control (Costa et al., 2008; Martin-Rhee and Bialystok, 2008), updating (Morales et al., 2013), and switching (Bialystok and Shapero, 2005; Bialystok and Viswanathan, 2009), bilingualism may also influence performance on planning skills in children. Therefore, in the current study, we examined the effects of bilingualism on planning abilities in school-aged children.

### Planning in Monolingual and Bilingual Children

Planning skills have traditionally been measured using Tower-type tasks (Simon, 1975; Shallice, 1982). For example, in the Tower of London (ToL) task, participants are presented with two arrangements of beads on pegs. The basic version of the task uses three differently colored beads on three pegs of different lengths. Participants are asked to match the first arrangement (termed initial-state) of beads to the second arrangement (termed goal-state) through a restricted pattern of movements. Importantly, participants are encouraged to plan their steps for solving the problem *before* making the first move. Due to the complex nature of planning, several cognitive processes have been implicated in planning abilities. For example, inhibitory control plays a crucial role in planning (Goel and Grafman, 1995; Welsh et al., 1999; Zook et al., 2004; Asato et al., 2006), where prepotent responses need to be inhibited to avoid making excessive moves, and moves leading to an incorrect solution need to be suppressed. Similarly, working memory or updating is also involved in planning (Phillips et al., 1999; Miyake et al., 2000) where the rules of the task need to be maintained and updated as moves are made in the direction of the goal-state configuration. Finally, shifting skills are associated with planning (Bull et al., 2004), as individuals must switch among sub-goals or moves to achieve the final configuration.

Extensive, and in recent years quite contentious, research has examined whether bilinguals demonstrate advantages on non-verbal EF tasks. For instance, while there is a significant body of knowledge that has suggested superior bilingual performance on inhibitory control tasks (Bialystok et al., 2004; Colzato et al., 2008; Costa et al., 2008; Martin-Rhee and Bialystok, 2008), evidence also exists indicating null effects of bilingualism on non-verbal inhibitory control skills (Bialystok et al., 2005; Hilchey and Klein, 2011; Paap and Greenberg, 2013; Antón et al., 2014; Duñabeitia et al., 2014). Similarly, with respect to non-verbal updating and shifting, while some studies have demonstrated positive effects of bilingualism (Bialystok and Martin, 2004; Prior and MacWhinney, 2010; Hernández et al., 2012; Morales et al., 2013), others have reported null results (Bonifacci et al., 2011; de Abreu et al., 2012; Paap and Greenberg, 2013; Gathercole et al., 2014).

In the vast majority of prior studies examining bilingual effects on EF, simple measures of the various EF components (inhibition, shifting, updating) have been used [but see Antón et al. (2014) who used an Attentional Network Test task, a complex task that combines elements of a flanker task with cueing]. However, there is significant evidence that simple measures of EF that purportedly measure the same component (e.g., inhibition) do not converge with each other (Stins et al., 2005; Humphrey and Valian, 2012; Paap and Greenberg, 2013), and that performance on simple EF tasks (e.g., the Simon task) are highly sensitive to task parameters, including the number and the timing of the stimuli (Valian, 2015). In the present study, we considered the possibility that complex EF tasks like planning – which rely on multiple components of EF and which may be less susceptible to the task-internal parameters and more susceptible to strategic, top-down influences (Miyake et al., 2000) – may be sensitive to the effects of bilingualism, especially in childhood. Our focus on planning was also conditioned by the relatively unexamined issue of bilingual children’s performance on planning tasks, since empirical work on bilingual planning has been sparse and primarily focused on adults (Craik and Bialystok, 2006; Festman et al., 2010; Penn et al., 2010).

The first study to examine planning performance in bilinguals was conducted by Festman et al. (2010) and involved a Tower of Hanoi task. The Tower of Hanoi task is a variation of the ToL task that uses disks instead of beads, and pegs that vary in their diameter rather than length. Bilingual adults were divided into switchers and non-switchers based on the number of cross-language errors made on a picture naming task. The switchers represented bilinguals with poor language control, while the non-switchers represented bilinguals with good language control. The authors found that the non-switchers demonstrated fewer errors on the Tower of Hanoi compared to the switchers, suggesting that bilinguals with stronger language control are also better at planning. In another study examining bilingual planning, Craik and Bialystok (2006) administered a “cooking breakfast” planning task to younger and older monolingual and bilingual adults. Participants had to remember to start and stop cooking five different foods so that all five dishes were ready at the same time. The authors found superior planning performance in older bilinguals than in monolinguals. Finally, Penn et al. (2010) tested bilingual and monolingual adults with aphasia on the ToL task. Results revealed that bilingual participants with aphasia performed within normal limits of the planning task, and significantly better than monolingual participants with aphasia, who demonstrated planning deficits.

Together, these findings indicate that bilingualism may be associated with enhanced planning abilities in adulthood. However, in a recent study, Jalali-Moghadam and Kormi-Nouri (2015) demonstrated that bilingualism played no role in planning performance in *children*. Jalali-Moghadam and Kormi-Nouri (2015) tested monolingual and bilingual children (9–12 years old) with and without reading difficulties on a battery of EF tasks, including the Tower of Hanoi. Analyses revealed *no* differences in planning performance between bilinguals and monolinguals. However, children with reading difficulties performed less well than the control groups. Thus, the work on bilingual planning is very sparse, and the one study of bilingual planning in children indicated a stronger role of language ability^1^ than of bilingualism in planning performance. Therefore, in the present study, we aimed to examine planning abilities in bilingual and monolingual children with the view to identify the role of language in bilingual and monolingual children’s planning performance.

### The Role of Language in Planning

Children often produce private speech during their preschool years to regulate their thought and behavior. Overt private speech gradually shifts to covert private speech during middle childhood (Vygotsky, 1987; Winsler, 2009; Lidstone et al., 2010). Famously, Vygotsky (1987) stated that by middle childhood, goal-directed thinking and self-regulation are fundamentally verbal in nature, being mediated by online self-directed speech – a process now termed *verbal mediation*. Verbal mediation appears to allow children to conceptualize the higher-order rules required for completing EF tasks, thereby facilitating EF performance (Zelazo et al., 1997). A number of studies have documented a link between verbal mediation and EF performance (Behrend et al., 1992; Winsler et al., 1997, 2007; Müller et al., 2004, 2008), including planning specifically (Fernyhough and Fradley, 2005; Al-Namlah et al., 2006). Experimental studies aimed at delineating the role of self-directed language in EF performance typically employ verbal suppression paradigms.

The logic behind verbal suppression is that if verbal mediation is used during a task, then interfering with the use of language should impede task performance. To prevent verbal mediation, a dual-task paradigm is implemented where a secondary task that is verbal in nature is performed simultaneously with the primary task. If performance costs are observed on the primary task, then the secondary task is hypothesized to share verbal demands with the primary task. A secondary task employed by prior studies to test verbal mediation is articulatory suppression where participants are asked to repeat a word or a sequence of words while completing the primary task. A number of studies have demonstrated that preventing the use of language through articulatory suppression *does* interfere with performance on EF tasks (Baddeley et al., 2001; Baldo et al., 2005; Whitehouse et al., 2006; Ang and Lee, 2008), and with planning in particular (e.g., Wallace et al., 2009; Lidstone et al., 2010, 2012). For example, Wallace et al. (2009) found that typically developing adolescents took significantly more moves to complete ToL problems under articulatory suppression than without articulatory suppression, indicating that planning was facilitated by verbal mediation and that verbal suppression disrupted planning performance.

One complication with interpreting the results of prior studies examining the role of verbal mediation in EF performance using articulatory suppression is that these studies have rarely included a control condition where the secondary task was non-verbal in nature. Therefore, it is not clear whether EF performance under articulatory suppression is in fact due to the suppression of verbal mediation or simply to the imposition of a secondary task. To remedy this issue, Emerson and Miyake (2003) introduced a comparable secondary task control condition to articulatory suppression that was non-verbal – foot tapping. In this motor-suppression condition, participants were asked to tap their foot to a metronome beat, similar to the articulatory suppression condition where they were asked to say “A-B-C” out loud to a metronome beat. The study revealed that articulatory suppression and foot tapping affected the adults’ performance on a visual EF task equally, both being worse than the baseline (no dual task) condition.

In children, only two studies examined the role of verbal mediation in planning by contrasting planning performance in the presence of a verbal vs. a non-verbal secondary task (Lidstone et al., 2010, 2012). Lidstone et al. (2010) tested children between 7 and 10 years of age on the ToL with articulatory suppression and motor suppression, and Lidstone et al. (2012) tested typically developing school-age children and children with Specific Language Impairment between 7 and 11 years of age on the same paradigm. Both studies showed that all children suffered performance costs on the ToL task in the articulatory suppression condition compared to the motor suppression condition, especially when children were asked to plan ahead (Lidstone et al., 2010). The authors concluded that suppression of verbal mediation, and not the imposition of a dual task, impeded planning abilities in children.

In summary, positive effects of bilingualism on planning abilities have been observed in adulthood (Craik and Bialystok, 2006; Festman et al., 2010) but not in childhood (Jalali-Moghadam and Kormi-Nouri, 2015). Verbal mediation appears to be important for planning performance (Fernyhough and Fradley, 2005; Lidstone et al., 2010); yet, the role of verbal mediation in relation to bilingual vs. monolingual planning has not been examined. The goal of the present study therefore was to examine planning abilities in bilingual vs. monolingual children focusing on the contributions of verbal mediation to planning in the two groups.

### Current Study

In the present study, we administered the ToL task to assess planning skills in 9–12-year-old English-speaking monolingual children and in Spanish–English simultaneous bilingual school-aged children matched on age and non-verbal intelligence. The age range of 9–12 years was chosen because it is during this period that children’s planning abilities appear to improve the most. Although even 3-year-old children can verbalize plans for familiar events (Hudson et al., 1995), complex planning through use of strategic reasoning matures between the ages of 9 and 12–13 years (Welsh et al., 1991; Anderson et al., 1996) and continues to improve though adolescence and early adulthood (Levin et al., 1991; De Luca et al., 2003; Romine and Reynolds, 2005).

Our focus on Spanish–English bilingual children was conditioned by the fact that Hispanic children represent the largest bilingual population in the United States (U.S. Census Bureau, 2016). In general, Hispanic children tend to occupy households characterized by lower SES than Caucasian children (Camarota, 2012), and these discrepancies in SES between the two populations are often cited as possible reasons for the inconsistency in the bilingual EF literature (Paap et al., 2015). Because SES is strongly linked with EF development [see review by Hackman et al. (2010)], it is possible that when bilingual children are characterized by higher SES than their monolingual peers, the effects of SES and bilingualism on EF become confounded. However, the inverse is very unlikely to hold – that is, given that SES affects EF negatively, bilingual children characterized by lower levels of SES should not outperform their higher SES monolingual peers. The present study, where the simultaneous bilingual children were indeed characterized by lower levels of SES than the monolingual children, provides a very stringent test of bilingual effects on planning. Finding planning advantages in our bilingual group would suggest that bilingualism may offset the EF disadvantages associated with lower SES. The following specific research questions were asked:

*First*, we asked whether differences in language experience would influence children’s performance on the ToL task. We hypothesized that if bilingualism enhances planning, then bilingual children should outperform monolingual children on the ToL task. In testing this question, we examined planning performance over time, comparing bilingual and monolingual children’s performance on early trials vs. late trials. Prior studies have indicated that planning performance improves with practice (Unterrainer et al., 2003), likely because participants learn how to generate and apply successful planning strategies with experience. If bilingualism facilitates the ability to generate successful planning strategies, then the effects of bilingualism on planning may be particularly strong for early trials, but dissipate with time as all children gain experience with the task.
*Second*, we examined the role of verbal mediation in planning by implementing a dual-task paradigm. All children completed three sets of ToL problems: one with no secondary task (baseline; NST), one with articulatory suppression task (AST), and one with motor suppression task (MST). We hypothesized that if verbal mediation *specifically* contributes to successful planning, planning performance should be more negatively affected by articulatory suppression than by motor suppression. Here, we were particularly interested in examining whether monolingual and bilingual children would perform differently on the ToL task under articulatory suppression and under motor suppression.

## Materials and Methods

### Participants

Sixty-five monolinguals and 56 simultaneous bilinguals between the ages of 9 and 12 years were recruited from local schools in Madison, WI, United States. Monolingual children spoke English as their native language; exposure to any language other than English was an exclusionary criterion. Bilingual children spoke both English and Spanish; exposure to a third language (defined as >5% during the week) was an exclusionary criterion. In-person interviews were conducted with the parents of bilingual children regarding their child’s language acquisition history and exposure. Exclusionary criteria for all the children included a diagnosis of language impairment, learning disability, psychological/behavioral disorders, neurological impairments, or other developmental disabilities. All children passed a hearing screening at 20 dB at 1000, 2000, and 4000 Hz.

Children from the two participant groups were selected to match on age and non-verbal IQ, resulting in 44 monolingual children (all reported to be non-Hispanic; 24 females) and 44 simultaneous bilingual children (35 reported to be Hispanic; 24 females). All bilingual children were simultaneous bilinguals who acquired both languages at or before the age of 3 years and were characterized by fairly balanced exposure to English and Spanish on a weekly basis (57.19% to English and 42.76% of the time to Spanish). Total years of maternal education were used as a proxy for socioeconomic status (SES). Although maternal education level may not capture all the nuances of SES, it has been widely and reliably used to approximate SES (e.g., Hoff, 2013). The bilingual children in the current study were significantly lower in SES than the monolingual children (*p* < 0.001).

All the children in the study were right-handed (per parent report) and used their right hand to perform the ToL task.

### Standardized Measures

Non-verbal intelligence was measured using the Perceptual Reasoning Index of the Wechsler Intelligence Scale for Children, 4th Edition (WISC-IV; Wechsler, 2003). The index consists of scores from three different subtests of the WISC – Block Design, Picture Concepts, and Matrix Reasoning. English language skills for all children were evaluated by administering the Clinical Evaluation of Language Fundamentals – 4th Edition (CELF-4; Semel et al., 2003). Scores were obtained for core language, receptive language, and expressive language scales.

Spanish language skills for bilingual children were evaluated by administering the Spanish Edition of the CELF-4 (Semel et al., 2006). Bilingual children performed less well than monolingual children on Core, Receptive, and Expressive indexes of English knowledge (*p*-values < 0.05). Within the bilingual group, children performed better on English than Spanish Core, Receptive, and Expressive indexes of the CELF-4 (*p*-values < 0.05). See **Table 1** for demographic characteristics of the participants in the two groups and **Table 2** for language characteristics of the bilingual children.

**Table 1:** Participant characteristics.

	Monolingual (*n* = 44)	Bilingual (*n* = 44)
Age (years)	10.19 (0.90)	10.19 (0.94)
Non-verbal IQ (Perceptual Reasoning Index, WISC-IV)	111.91 (11.33)	111.68 (12.28)
Socioeconomic status (SES)^a^	17.67 (2.92)	14.21 (4.52)^∗∗∗^
English core language (CELF-4)	110.74 (11.94)	98.05 (16.36)^∗∗∗^
English receptive language (CELF-4)	111.73 (13.17)	103.50 (17.21)^∗^
English expressive language (CELF-4)	111.37 (12.44)	97.52 (17.09)^∗∗∗^

*The data represent means and standard deviations. The means for standardized measures represent standard scores. ^*a*^SES was indexed by total maternal years of education. ^∗^Significance level < 0.05. ^∗∗∗^Significance level ≤ 0.001.*

**Table 2:** Bilingual characteristics.

	*n* = 44
Spanish core language (CELF-4)	85.75 (14.24)
Spanish receptive language (CELF-4)	96.36 (11.15)
Spanish expressive language (CELF-4)	81.57 (15.64)
Current English exposure (%)^a^	57.19 (18.20)
Current Spanish exposure (%)^b^	42.76 (18.22)
English age of acquisition (months)^c^	7.75 (13.19)
Spanish age of acquisition (months)^d^	4.66 (12.91)
First language^e^	English – 43%; Spanish – 46%; both – 11%
Dominant language^f^	English – 68%; Spanish – 18%; both – 14%

*The data represent means and standard deviations. The means for standardized measures represent standard scores. ^*a*^English exposure = [(number of hours of English heard on a weekday ^∗^ 5 days per week) + (number of hours of English heard on Saturday and Sunday)]/(number of hours child is awake per week). ^*b*^Spanish exposure = 100 – percent of English exposure. The formula was adjusted to take into consideration special circumstances such as if a child heard one language more on certain days of the week. ^*c*^Parent report: age when child started hearing English on a daily basis by family members/caregiver. ^*d*^Parent report: age when child started hearing Spanish on a daily basis by family members/caregiver. ^*e*^Parent report: child’s first language. ^*f*^Parent report: child’s dominant language.*

### Procedure

Children were tested on standardized assessments and experimental tasks over the course of two-to-three 2-h visits to the laboratory. Trained bilingual research assistants administered the Spanish measures to the bilingual children.

#### Experimental Planning Task

Planning ability for all children was evaluated by a computerized version of the ToL task. This task was adapted for computerized presentation from the original version developed by Shallice (1982). In this version of the task, children were able to move the beads using the mouse, with minimal experimenter interference. The task was programed using the ToL software (Sanzen Neuropsychological Assessment Tests LLC, 2012), which allowed for generating problem sets (trials) depending on the desired number of pegs, beads, or number of moves. Only four-move problems, i.e., problems that can be solved in exactly four moves, with three pegs and three differently colored beads were selected. This level of difficulty was chosen so that the task would be challenging enough but not too complex for children in the target age range (Kaller et al., 2008). The trials were normed on adults to ensure that the difficulty levels of the problems were comparable. Twenty-two four-move problems were administered to 28 college undergraduate students. Trials that took the greatest number of moves and most time to complete were eliminated (seven trials). The final stimulus set consisted of 15 trials that were equivalent in difficulty level where the number of moves ranged from 4 to 4.76 and total time to complete ranged from 7.79s to 13.17s.

The 15 trials were randomly assigned to three task conditions –NST, AST, and MST. Each condition included five trials, with the same five trials presented to all children. However, the conditions were presented in randomized order for each participant, and the order of the trials within each condition was pseudorandomized. In the NST condition, children completed the ToL task without any secondary task demands. In the AST condition, children were instructed to say the word “maybe” out loud while performing the ToL task. Children said “maybe” every time they heard a beep, which was generated by E-Prime Studio 2.0. The beeps consisted of repeated presentations of a simple tone every 750 ms. Children’s verbal responses were recorded using a digital recorder. In the MST condition, children were asked to tap their foot on a pedal while performing the ToL task. All children used their right foot to press the pedal. Similar to the AST condition, children tapped their foot every time they heard a beep. Foot pedal responses were recorded by E-prime. Participants were redirected to continue the secondary task (tapping the foot or saying “maybe”) if they forgot to do so during the experiment. Prior to the experimental session, children were administered five untimed ToL practice trials without any secondary tasks with appropriate verbal feedback. The practice trials did not appear in the experimental conditions.

**FIGURE 1:**
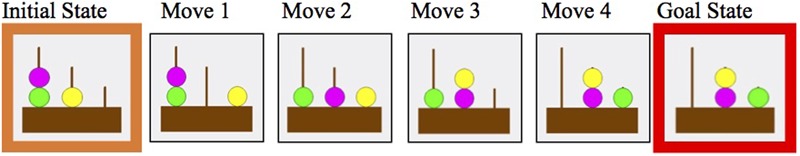
The ToL task. Example of a practice ToL trial showing the initial state position and the goal state position. Beads were red, blue, and green for test trials. All trials were four-move problems.

On each ToL trial, participants were presented with two arrangements of beads on pegs on the computer screen and were asked to move the beads in one arrangement – Picture 1 – to match the other arrangement – Picture 2 (see **Figure 1** for a visual depiction of the task). Children were explicitly told to think about how they would match the pictures before they moved the first bead. A 2-s inter-stimulus fixation point was presented between each trial. The task yielded precise accuracy and reaction time (RT) measures. Children’s performance on the ToL task was measured by *number of moves* (total moves made to complete a trial), *planning time* (time taken to move the first bead), and *execution time* (time taken to complete the trial after the first bead was moved). If the child completed a trial successfully, i.e., matched Picture 1 to Picture 2 in under 20 moves or under 75 s, the trial was retained for the analyses. If a child made more than 20 moves or exceeded the time limit of 75 s from trial onset, the experiment proceeded to the next trial, and the trial was eliminated from data analyses. A total of 0.02% of trials were eliminated.

Performance on the secondary tasks was measured in order to examine whether the difficulty levels of the two secondary tasks differed from each other. MST performance was calculated by taking a proportion of the number of foot presses to the total number of beeps presented during the condition. AST performance was calculated by taking a proportion of the number of “maybes” said by the child to the total number of beeps presented during the condition.

### Analyses

For the ToL task, trials in each condition were split into an early phase (trials 1 and 2) and a late phase (trials 3, 4, and 5) to examine learning effects, if any. Mixed effects models using the lme4 package (Bates et al., 2015) in R were run separately for each of the three dependent variables: number of moves, planning time, and execution time. Each model included the fixed effects of group (monolingual vs. bilingual), condition (NST vs. MST vs. AST), and phase (early vs. late), and the two-way interactions between group and condition and between group and phase. Models also included the covariate SES quantified by the maternal years of education, although it should be noted that the reported significant effects were also significant when SES was not covaried.^2^ A random by-subject intercept and random by-subject slopes for condition and phase were also included.

Performance on secondary tasks was not split into early and late trials and therefore the model did not contain any fixed or random effects related to phase. The variables group and phase were centered and contrast coded as -0.5 and 0.5 (monolingual, bilingual; early, late). Condition was coded initially with the reference group NST and the reference group was changed and the model was re-run if a significant effect of condition was observed. *t-*Values > 1.96 were considered significant at *p* < 0.05.

Parallel to our regression analyses, we ran a Bayesian factor analysis using the BayesFactor package (Morey and Rouder, 2015) examining the probability that our data favored the alternative hypothesis. For each regression analysis, we also report the Bayes factor which is the likelihood that our data favor the null hypothesis. Smaller numbers (<1) are in favor of the alternative hypothesis.

## Results

### Number of Moves

See **Table 3** for a representation of the raw data for number of moves. A Wald test revealed that the addition of condition significantly improved the model (χ^2^(2) = 14.00, *p* < 0.001) such that children made significantly fewer moves in the NST (*M* = 4.65, *SE* = 2.04) condition than the MST (*M* = 5.23, *SE* = 2.65; *b* = 0.58, *SE* = 0.18, *t* = 3.23, *p* < 0.05) or the AST (*M* = 5.31, *SE* = 3.18; *b* = 0.66, *SE* = 0.21, *t* = 3.16, *p* < 0.05) conditions. Number of moves in the MST and AST conditions did not significantly differ (*b* = 0.08, *SE* = 0.20, *t* = 0.43, *p* > 0.05). The addition of group (χ^2^(1) = 0.59, *p* > 0.05), phase (χ^2^(1) = 0.44, *p* > 0.05), the interaction between group and condition (χ^2^(2) = 0.10, *p* > 0.05), and the interaction between group and phase (χ^2^(1) = 0.35, *p* > 0.05) did not significantly improve the model. See **Table 4** for the full regression model with NST as the reference group.

**Table 3:** Performance on the ToL.

Condition	Monolingual		Bilingual	
	Early	Late	Early	Late
**Number of moves**				
NST	4.62 (2.11)	4.58 (2.34)	4.56 (1.28)	4.79 (2.09)
MST	5.46 (3.15)	4.91 (2.20)	5.39 (2.86)	5.28 (2.55)
AST	4.81 (2.03)	5.45 (3.43)	5.17 (2.88)	5.59 (3.67)
**Planning times (seconds)**				
NST	5.90 (3.89)	4.70 (2.85)	4.33 (2.14)	3.97 (1.94)
MST	6.47 (4.04)	4.93 (2.81)	5.24 (3.21)	3.95 (1.62)
AST	4.92 (2.44)	4.00 (2.15)	4.09 (2.07)	3.87 (1.62)
**Execution times (seconds)**				
NST	9.01 (5.83)	9.09 (8.29)	8.98 (4.08)	9.31 (7.75)
MST	15.62 (10.99)	12.82 (9.36)	15.64 (11.16)	14.89 (11.24)
AST	10.89 (7.62)	11.88 (10.27)	11.11 (9.68)	11.87 (10.79)

*The data represent means and standard deviations.*

**Table 4:** Regression models with NST as reference group.

	*b*	*SE*	*t*
Number of moves			
Intercept	4.64	0.12	37.17*
SES	-0.05	0.09	-0.56
Group	0.06	0.25	0.26
Condition-AST	0.66	0.21	3.16*
Condition-MST	0.58	0.18	3.23*
Phase	0.10	0.15	0.67
Group × Condition-AST	0.12	0.42	0.28
Group × Condition-MST	0.09	0.36	0.25
Group × Phase	0.18	0.30	0.59
Planning times
Intercept	4.75	0.19	24.79*
SES	0.08	0.13	0.61
Group	-1.11	0.39	-2.87*
Condition-AST	-0.49	0.20	-2.50*
Condition-MST	0.37	0.24	1.52
Phase	-0.92	0.14	-6.67*
Group × Condition-AST	0.68	0.39	1.72
Group × Condition-MST	-0.04	0.49	-0.08
Group × Phase	0.58	0.28	2.09*
Execution times
Intercept	9.14	0.45	20.16*
SES	-0.14	0.33	-0.42
Group	2.43	0.66	3.70*
Condition-AST	5.45	0.65	8.36*
Condition-MST	-0.001	0.92	-0.001
Phase	-0.24	0.51	-0.47
Group × Condition-AST	-0.08	1.32	-0.06
Group × Condition-MST	1.16	1.30	0.02
Group × Phase	0.74	1.01	0.73
Secondary tasks
Intercept	0.78	0.02	49.26*
SES	0.02	0.01	1.83
Group	0.03	0.03	1.04
Condition-AST	0.28	0.03	10.37*
Condition-MST	0.001	0.05	0.03

*^∗^Significance level < 0.05.*

A Bayesian factor analysis confirmed that our data were in favor of the alternate hypothesis (BF_01_ = 0.04). That is, our data are 23.8 times more likely under a model that includes the effect of condition than a model without it. Similarly to our frequentist regression results, the Bayes factors for all other variables in the model were more in favor for a model without group (BF_01_ = 3.33), without phase (BF_01_ = 4.61), without the interaction between condition and group (BF_01_ = 32.62), and without the interaction between group and phase (BF_01_ = 4.83).

### Planning Time

See **Table 3** for a representation of the raw data for planning time and **Table 4** for the full regression model. The addition of condition significantly improved the model (χ^2^(2) = 28.32, *p* < 0.001) such that planning time for the AST condition (*M* = 4.16, *SE* = 2.08) was significantly shorter than for the NST (*M* = 4.65, *SE* = 2.82; *b* = 0.49, *SE* = 0.20, *t* = 2.50, *p* < 0.05) and MST conditions (*M* = 5.00, *SE* = 3.02; *b* = 0.86, *SE* = 0.17, *t* = 4.99, *p* < 0.05). The NST and MST conditions did not significantly differ from each other (*b* = 0.37, *SE* = 0.24, *t* = 1.52, *p* > 0.05). The effect of group also significantly improved the model (χ^2^(1) = 5.59, *p* < 0.05), where bilinguals (*M* = 4.17, *SE* = 2.12) overall took less planning time than monolinguals (*M* = 5.03, *SE* = 3.11). The addition of phase also improved the model (χ^2^(1) = 44.31, *p* < 0.001), such that earlier trials (*M* = 5.15 *SE* = 3.16) required longer planning time than late trials (*M* = 4.23, *SE* = 2.25).

The interaction between group and condition was significant (χ^2^(2) = 6.43, *p* < 0.05). Follow-up between-group analyses revealed that monolinguals had significantly longer planning times than bilinguals for the NST (*b* = 1.11, *SE* = 0.39, *t* = 2.87, *p* < 0.05) and MST conditions (*b* = 1.15, *SE* = 0.39, *t* = 2.92, *p* < 0.05) but the groups did not differ in planning time in the AST condition (*b* = 0.43, *SE* = 0.30, *t* = 1.46, *p* > 0.05). Follow-up within-group analyses revealed that for monolinguals, the AST condition led to significantly shorter planning times than the NST (*b* = 0.83, *SE* = 0.28, *t* = 2.98, *p* < 0.05) and the MST (*b* = 1.22, *SE* = 0.24, *t* = 4.98, *p* < 0.05) conditions, and the NST and MST conditions did not differ (*b* = 0.38, *SE* = 0.35, *t* = 1.13, *p* > 0.05). For bilinguals, the planning times in the AST condition were significantly shorter than in the MST condition (*b* = 0.50, *SE* = 0.24, *t* = 2.07, *p* < 0.05) but not the NST condition (*b* = 0.15, *SE* = 0.28, *t* = 0.55, *p* > 0.05), and NST and MST conditions did not differ from each other (*b* = 0.35, *SE* = 0.35, *t* = 1.02, *p* > 0.05).

Finally, the interaction between group and phase was also significant (χ^2^(1) = 4.38, *p* < 0.05) such that monolinguals had significantly longer planning times than bilinguals for the early trials (*b* = 1.40, *SE* = 0.43, *t* = 3.23, *p* < 0.05), but not the late trials (*b* = 0.82, *SE* = 0.39, *t* = 2.13, *p* > 0.05). Within-groups follow-up analyses revealed that the decrease in planning time with later trials was significantly greater for monolinguals (*b* = 1.21, *SE* = 0.20, *t* = 6.18, *p* < 0.05) than for bilinguals (*b* = 0.63, *SE* = 0.19, *t* = 3.24, *p* < 0.05).

The Bayes factor analysis revealed that our data were 4032 times more likely under a model that included condition than a model without condition (BF_01_ = 2.48E-4). Our data were also 18.76 times more likely under a model with the effect of group than without it (BF_01_ = 0.053) and 303 million times more likely under a model with phase than without it (BF_01_ = 3.38E-9). Unlike our frequentist analysis, the Bayes analysis was in favor of a model which did not include the interaction between condition and group (BF_01_ = 2.89), and there was only mild support for the inclusion of the interaction between group and phase (BF_01_ = 0.84).

### Execution Time

See **Table 3** for a representation of the raw data for execution time. In order to achieve model convergence, the random by-subject effect of phase was removed. The addition of condition significantly improved the model (χ^2^(2) = 70.33, *p* < 0.001), such that the execution time for the NST condition (*M* = 9.12, *SE* = 6.94) was significantly shorter than that for the MST (*M* = 14.56, *SE* = 10.67; *b* = 5.45, *SE* = 0.65, *t* = 8.36, *p* < 0.05) and AST conditions (*M* = 11.53, *SE* = 9.83; *b* = 2.43, *SE* = 0.66, *t* = 3.70, *p* < 0.05). Furthermore, execution time for the MST condition was significantly longer than for the AST condition (*b* = 3.02, *SE* = 0.73, *t* = 4.13, *p* < 0.05). The addition of group (χ^2^(1) = 0.20, *p* > 0.05), phase (χ^2^(1) = 0.22, *p* > 0.05), the interaction between group and condition (χ^2^(2) = 0.98, *p* > 0.05), and the interaction between group and phase (χ^2^(1) = 0.53, *p* > 0.05) did not significantly improve the model. See **Table 4** for the full regression table with NSF as the reference group.

A Bayesian factor analysis confirmed that our data were 5.64E+13 times more likely in a model which included condition than one that did not (BF_01_ = 1.77E-14). However, similar to our frequentist analysis, our Bayesian factors were more in favor of a model that did not include group (BF_01_ = 3.47), phase (BF_01_ = 5.12), the interaction between group and condition (BF_01_ = 17.51), or the interaction between group and phase (BF_01_ = 4.44).

### Performance on Secondary Tasks

See **Table 5** for a representation of the raw data for accuracy on the secondary tasks. The average number of taps or “maybe” as a proportion of total number of beeps heard was regressed on the interaction between secondary task type (AST vs. MST) and group. Analyses revealed that the addition of task type significantly improved the model (χ^2^(1) = 107.51, *p* < 0.05), such that the children were more accurate for the AST (*M* = 0.92, *SE* = 0.10) than the MST (*M* = 0.65, *SE* = 0.23) condition. The effect of group (χ^2^(1) = 2.09, *p* > 0.05) and the interaction between group and condition (χ^2^(1) = 0.001, *p* > 0.05) did not significantly improve the model. See **Table 5** for the full regression model.

**Table 5:** Performance on Secondary Tasks in Results.

	Monolingual	Bilingual
MST	0.65 (0.27)	0.65 (0.20)
AST	0.91 (0.11)	0.92 (0.09)

*The data represent means and standard deviations. The means represent the average number of taps or “maybe” as a proportion of total number of beeps heard.*

The Bayesian approach similarly revealed that our data were 2.43E+16 times more likely to occur in a model that included the effect of condition than one without it (BF_01_ = 4.11E-17). Our data were also more likely to occur in a model that did not include group (BF_01_ = 3.57) or the interaction between group and condition (BF_01_ = 6.02).

## Discussion

The purpose of this study was to examine the effects of bilingualism on planning abilities, as measured by the ToL task. We found that bilingual children were faster planners than monolingual children, but only for the early trials of the planning task. Furthermore, these group differences in planning times were only observed in the absence of the secondary task and when the secondary task involved motor suppression (but not articulatory suppression). Finally, compared to motor suppression, articulatory suppression did not disrupt ToL performance on any of the measures for either participant group.

### Group Differences in Planning Performance

We found that bilingual children had faster planning times (time to move the first bead) than monolingual children. However, the two groups did not differ in total number of moves or execution times for the task. Therefore, the shorter planning times in bilinguals did not negatively affect their performance on other aspects of the task, suggesting that bilingual children were more efficient at planning than monolingual children. This finding is consistent with prior work in adults (Craik and Bialystok, 2006; Festman et al., 2010) but it conflicts with findings from Jalali-Moghadam and Kormi-Nouri (2015) who found no performance differences between bilingual and monolingual children on the Tower of Hanoi task.

One difference between our study and the Jalali-Moghadam and Kormi-Nouri (2015) study is how the variables of interest were defined. Jalali-Moghadam and Kormi-Nouri (2015) defined their measures of performance in terms of number of moves and the *total* time taken to complete the task. In the present study, we divided the time variable into two parts: time taken to make the first move (defined as planning) and time taken to complete the task thereafter (defined as execution). Although our results aligned with their findings in terms of number of moves, differences in how efficiency was measured is the likely reason for the discrepant results with regard to time. Time to make the first move is an important measure of Tower performance when participants are told to preplan (Phillips et al., 2001; Unterrainer et al., 2003; Berg et al., 2010).

Another difference between our study and that of Jalali-Moghadam and Kormi-Nouri (2015) is that Jalali-Moghadam and Kormi-Nouri (2015) administered the Tower of Hanoi task whereas we administered the ToL task to index planning. While the ToH task shares some features with the ToL task, differences in the set-up and instructions may contribute to different task demands. In fact, Bull et al. (2004) demonstrated that the ToL and the Tower of Hanoi are not interchangeable. In addition to differences in surface characteristics, ToH is more strongly associated with mental flexibility while ToL has stronger links with inhibition in children (Bull et al., 2004). Therefore, the recruitment of different executive processes may alter the strategies that children use to complete the two planning tasks. Further, instructions to the tasks might yield different expectations for the child, such that they influence whether children plan ahead of time (like in Lidstone et al., 2010) or resort to more online perceptual planning (like in Jalali-Moghadam and Kormi-Nouri, 2015).

A final difference between our study and Jalali-Moghadam and Kormi-Nouri (2015) is the difficulty level of the planning tasks. Jalali-Moghadam and Kormi-Nouri (2015) used seven-move problems, whereas we used four-move problems, which are easier (Kaller et al., 2012). The increased difficulty of the task might also have rendered group differences non-significant in the Jalali-Moghadam and Kormi-Nouri (2015) study. Prior studies suggest that as planning tasks become more difficult, children do not necessarily plan ahead of time, but instead have bursts of planning during the task itself (Hayes-Roth and Hayes-Roth, 1979; Phillips et al., 1999), especially when a secondary task is imposed (Phillips et al., 1999). With increased complexity, children may use a strategy of online planning since it is less demanding. Conversely, at easier levels, it is more conceivable to plan ahead. In the present study where the task was sufficiently difficult to yield variability in planning performance, and yet sufficiently easy to enable children to perform the task successfully, children may have been better able to plan ahead, and bilingual children were especially effective at planning ahead.

An important finding in the present study was that all children demonstrated phase effects in planning and were more likely to demonstrate lower levels of performance in the first two trials vs. the last three trials of the task. This suggests that all children adapted to the paradigm with practice, and may have learned to develop strategies to solve the problems. This supports previous research in adults showing that participants’ performance on the ToL task improved significantly in the second block of trials vs. the first block of trials (Unterrainer et al., 2003). The authors interpreted this finding as a demonstration of learning effects, where participants developed effective planning strategies in the course of practice. In the current study, bilingual advantages in planning times were only observed for the early trials, and group differences disappeared in the late trials. That is, both bilingual and monolingual children benefitted from practicing the task, but the benefit was much greater for the monolingual children who were able to catch up to the bilinguals. This indicates that the effects of bilingualism on planning were rooted in bilinguals’ superior initial ability to generate successful planning strategies.

The main finding that bilinguals were more efficient planners than monolinguals speaks to the broader issue of the presence of bilingual advantages in executive functioning. Previous work has yielded conflicting evidence regarding bilingual effects on EF, with some studies demonstrating positive effects of bilingualism (Costa et al., 2008; Prior and MacWhinney, 2010; Morales et al., 2013) and some showing null results (Paap and Greenberg, 2013; Paap et al., 2015). We believe that our finding of bilingual advantages on the ToL task may be the result of using a complex EF task that relies on multiple simple EF components (Miyake and Friedman, 2012). Future studies could extend this work by examining the effects of bilingualism on EF performance at different levels of task complexity. In the meantime, we would highlight that we found superior planning performance in bilingual children who were characterized by lower SES than their monolingual peers. The bilingual/monolingual difference in planning times maintained after controlling for SES. Since lower levels of SES would be expected to depress planning performance, our finding of planning advantages in this sample of bilinguals is strong evidence of bilingual effects on planning abilities indeed. The question is – *why* did bilingual children outperform monolingual children on the ToL task. By examining planning performance under articulatory vs. motor suppression conditions, we aimed to test whether self-directed use of language may be at the core of bilingual/monolingual planning differences.

### The Role of Verbal Mediation in Planning

Compared to the NST control condition, all children were adversely affected by AST for number of moves and execution times. For planning times, on the other hand, the AST condition yielded significantly faster RTs than the NST condition. Compared to the MST, children were not negatively affected by AST for number of moves, planning times, or execution times. In fact, AST planning times were significantly shorter than MST planning times.

Our results do not converge with findings from Lidstone et al. (2010, 2012) who showed that ToL performance was disrupted by articulatory suppression more so than by motor suppression. However, it is notable that other studies examining the effects of articulatory suppression on planning have produced results similar to ones we observed here. For instance, Phillips et al. (1999) showed that articulatory suppression did not impair performance on the ToL task in terms of the number of moves, and yielded shorter planning times compared to the control condition. The authors interpreted this finding to suggest that articulatory suppression discouraged the application of inefficient verbal strategies and promoted more effective visuospatial strategies. Brandimonte and Gerbino (1993) made a similar argument claiming that suppression of verbal mediation may improve performance on some visuospatial tasks, like the Reversible Images task.

There are some commonalities between our study and the Phillips et al. (1999) study that differentiate it from the Lidstone et al. (2010, 2012) studies. These comparisons lead us to interpret the results as suggesting that in our particular implementation of the ToL task, the use of verbal mediation may have been counterproductive. Specifically, the ToL task in Phillips et al. (1999) study and our study was computerized, whereas Lidstone et al. (2010, 2012) used a physical version of the ToL. A two-dimensional, visuospatial task presented on a computer might load more heavily on visual processing and less heavily on verbal mediation, than a three-dimensional, physically manipulatable task. Phillips et al. (1999) and others (Brandimonte and Gerbino, 1993; Hitch et al., 1995) have argued that verbal mediation may actually discourage efficient performance on some visuospatial tasks, and that verbal suppression can improve performance on such tasks because it encourages the use of visual code. Our findings are congruent with this interpretation of the verbal-suppression effect. One caveat to this interpretation is that our study also revealed that motor suppression was more detrimental to planning performance than articulatory suppression.

Children’s performance on the secondary tasks indicates that the two tasks were not equally demanding. Accuracy on the motor task was 65% while accuracy on the verbal task was 92%; that is, tapping the pedal with the foot was more taxing than saying “maybe.” This finding seemingly conflicts with results obtained by Emerson and Miyake (2003) who found that adults performed similarly on the Identical Pictures Test under motor suppression and articulatory suppression. However, Emerson and Miyake (2003) compared performance on the *primary task* only and did not compare adults’ performance on the two secondary tasks. Although performance on the primary task may be similar, the difficulty levels of the secondary tasks could still be non-equivalent. Case in point, in the current study, children did *not* differ in the number of moves they made under the AST and the MST conditions. However, performance on the secondary motor task was significantly lower than performance on the secondary verbal task. It is unclear why the motor secondary task was more difficult than the articulatory secondary task for the children in the present study. The two secondary tasks were likely both left-lateralized for the vast majority of the participants. That is, the left hemisphere of the brain was likely engaged by the secondary verbal task and the secondary motor task (since all children were right-handed and used their right foot to tap). Therefore, it is unlikely that differences in lateralization for the two secondary tasks contributed to the differences in their difficulty levels. However, future work utilizing similar dual-task paradigms should consider handedness and foot-dominance parameters in an effort to implement secondary tasks of equal difficulty. It is worth noting that designing equally difficult secondary tasks is a significant challenge, as the difficulty levels of the secondary tasks may be highly contingent on the parameters and the demands of the primary tasks.

A notable finding in our study with respect to articulatory suppression was that there was a significant effect of group membership on planning times, but only when the task allowed for verbal mediation to occur. That is, bilingual children outperformed monolingual children in the NST and MST conditions where language could have been used freely, but not in the AST condition, which presumably interfered with the ability to use language for planning purposes. It is possible that bilingual children were less likely to use verbal mediation for planning than monolingual children across the board, yielding the pattern of findings where group differences in planning were only significant for conditions that allowed verbal mediation. This interpretation is consistent with our interpretation of the finding that the AST condition yielded shorter planning times than the NST condition. That is, it may be that this version of the task was best approached through visual–spatial rather than verbal planning strategy, and bilingual children may have been more prone to this strategy than the monolingual children.

Whatever the reasons behind bilingual children’s performance, it is interesting to consider it in the context of bilingual children’s lower English language scores. Bilingual children tend to be less successful than monolingual children on language-specific tasks (Thordardottir et al., 2006; Gathercole and Thomas, 2009), likely because of reduced exposure and experience with language-specific information (Thordardottir et al., 2006). Bilinguals also often underperform relative to their monolingual peers on standardized language measures (Ben-Zeev, 1977; Hemsley et al., 2006; Uccelli and Paez, 2007; Vagh et al., 2009; Bialystok et al., 2010; Marchman et al., 2010; Hoff et al., 2012) and perform less successfully than monolinguals on processing tasks that require rapid access to language-specific knowledge (Ivanova and Costa, 2008; Bialystok and Feng, 2009; Costa, 2009). One hypothesis regarding the effects of lower levels of English ability on planning performance may be that it would be disadvantageous, since it might reduce the children’s ability to verbally mediate, at least in English. Our finding of bilingual advantages in planning indicates that lower English language skills did not compromise bilingual children’s planning performance. In the context of our ToL task, which may have encouraged the use of visual rather than verbal strategies, bilingual children’s lower English skills may have reduced their reliance on verbal mediation, and encouraged visual strategizing. However, future work should attempt to match bilingual and monolingual children on language abilities in an effort to examine whether bilingualism (or language skills or both) influences planning performance.

It is important to note here that it is unclear what exact features of language constitute verbal mediation and how (or even whether) language use during planning reflects performance on language measures. That is, the nature of verbal mediation in general is largely unknown, and how verbal mediation may be instantiated in bilingual speakers is even less clear. For instance, it may be that verbal mediation in bilinguals is language-specific, such that bilinguals rely on a particular language (e.g., the more dominant language or the language of the environment) for strategizing and planning during complex EF tasks. Conversely, it may be that just like general language processing in bilinguals is characterized by non-selectivity (Kroll et al., 2012; Kroll and Dussias, 2013), language use during complex EF tasks may also be non-selective, with children relying on both languages to verbally mediate planning. It would be important for future studies to undertake these questions, and to compare bilinguals’ performance on planning tasks under articulatory suppression in two different languages.

## Conclusion

We found a very circumscribed effect of bilingualism on children’s planning performance. Bilingual children were more efficient at planning than monolingual children, but only for an earlier phase of the planning task, only under certain conditions (NST and MST), and for only one outcome measure (planning times). How do we reconcile this finding with previous studies that have demonstrated bilingual advantages on EF tasks (Bialystok et al., 2004; Prior and MacWhinney, 2010; Morales et al., 2013) and with previous studies that have failed to do so (Paap and Greenberg, 2013; Antón et al., 2014; Gathercole et al., 2014)? We would suggest that our findings offer some insights into the possible reasons behind the highly conflicting literature on bilingual EF. The finding that the effect of bilingualism was only observed for the planning time measure (but not for any other measure) indicates that bilingual influences on EF performance are most likely to be obtained when the EF task involves strategic, top-down deployment of attention. Our finding that the positive effect of bilingualism was observed only for an earlier phase of the task indicates that bilingual advantages are more likely to emerge when the EF task under study is not over-practiced. This finding may also speak to the possibility that the effects of bilingualism are most likely to be revealed on an EF task whose difficulty level hits the “sweet spot” of being difficult enough to pose a challenge and yet not so difficult that high levels of accuracy cannot be attained. The finding that bilingual children outperformed monolingual children only under the NST and the MST conditions speaks less to the mechanisms by which bilingualism may influence EF performance, but rather indicates that performance on EF tasks (especially the complex EF tasks) may be exquisitely sensitive to task parameters.

We would however caution against over-interpreting the effects of articulatory suppression on planning performance in the present study in view of the fact that the two secondary tasks were not equivalent in their difficulty levels – the motor task was more difficult than the verbal task. Future work will need to carefully consider and operationalize the difficulty levels of the secondary tasks in relation to each other, and in the context of the primary task in order to move this line of inquiry further. A final qualification is that this study followed a two-group quasi-experimental design, which is a staple in bilingualism research, but also infamously precludes causal interpretations. With these caveats in place, our finding regarding group differences in planning performance do have important educational and practical implications.

The largest bilingual population in the United States is from Hispanic backgrounds (U.S. Census Bureau, 2016). The educational outcomes of Hispanic children tend to lag behind those of their non-Hispanic peers (Kao and Thompson, 2003; Kohler and Lazarín, 2007). Hispanic children have lower levels of school readiness at the start of kindergarten compared to White and Black children (Fryer and Levitt, 2004; Duncan and Magnuson, 2005), and this achievement gap remains largely unchanged through the late elementary and middle school years (Reardon and Galindo, 2009). Some researchers have suggested that differences in SES may contribute to the achievement gap (Fryer and Levitt, 2004), because in the United States, Hispanic children tend to come from lower SES households than non-Hispanic children (DeNavas-Walt et al., 2004). Others have attributed the achievement gap to differences in linguistic experience (Padilla and Gonzalez, 2001), where Hispanic children’s lower English proficiency might be a contributing factor to their lower levels of academic success. In the present study, the vast majority of the bilingual children were Hispanic, while the monolingual children were all non-Hispanic. Furthermore, the bilingual children in our study were characterized by lower levels of English language knowledge than the monolingual children, and by lower SES. Thus, our bilingual group is highly representative of the general Hispanic population of school-aged children in the United States. The results of the current study are therefore hopeful, in that they suggest that despite lower SES and English language skills, Hispanic children who are bilingual do not show deficits in planning compared to their non-Hispanic peers.

## Ethics Statement

This study was approved by the Education and Social/Behavioral Institutional Review Board at the University of Wisconsin-Madison, Protocol SE-2011-0818, Executive Function in Children with Typical and Atypical Language Abilities. Parents provided written consent after reading a description of the study and having an opportunity to ask questions. All children provided verbal assent.

## Author Contributions

SEW and MK conceived the study. IG was involved in the stimuli development, data collection, preliminary data analyses, and manuscript drafting. IG and MK interpreted the data. MM was involved in data analyses and writing of selected sections. IG, MM, SEW, and MK were involved in revising it critically for important intellectual content, provided the final approval of the version to be published, and agreed to be accountable for all aspects of the work in ensuring that questions related to the accuracy or integrity of any part of the work are appropriately investigated and resolved.

## Conflict of Interest Statement

The authors declare that the research was conducted in the absence of any commercial or financial relationships that could be construed as a potential conflict of interest.

## Footnotes

1 We infer the role of language in the Jalali-Moghadam and Kormi-Nouri (2015) study based on the close relationship between language and reading skills (Hugh et al., 2000; Catts and Kamhi, 2005).

2 We included SES but not English language ability (CELF Core scores) as a covariate because the two factors were highly correlated (*r* = 0.53, *p* < 0.001). Entering them both into the model would lead to multicollinearity, and thus to invalid standard errors and significance tests. Models run with CELF scores (instead of SES) as a covariate resulted in the same pattern of significance as models run with SES as a covariate. Models run with both CELF and SES as covariates also yielded largely the same pattern of results; however, as would be expected, the effect of group for the planning time became weaker (*p* = 0.05). Full models are available in Supplementary Materials.
