# Supplementary material for: Planning Abilities in Bilingual and Monolingual Children: Role of Verbal Mediation
Source: Front Psychol. 2018 Mar 14;9:323. doi: 10.3389/fpsyg.2018.00323 (PMC5861373; doi:10.3389/fpsyg.2018.00323)
Supplement: Supplementary file 1 [file Data_Sheet_1.docx]

Supplementary Material

Planning abilities in bilingual and monolingual children: Role of verbal mediation

**Ishanti Gangopadhyay^1,2*^, Margarethe McDonald^1,2^, Susan Ellis Weismer^1,2^, & Margarita Kaushanskaya^1,2^**

^1^Department of Communication Sciences and Disorders, University of Wisconsin-Madison, Madison, 53706, WI, USA

^2^Waisman Center, University of Wisconsin-Madison, Madison, 53705, WI, USA

*Correspondence:
Corresponding Author
Ishanti Gangopadhyay, Language Acquisition & Bilingualism Lab, 1500 Highland Avenue, Room 476, Madison, WI 53705. Email: [igangopadhya@wisc.edu](mailto:kaushanskaya@wisc.edu)

# Supplementary Tables

**Table 1a: Wald chi-squared test of model improvement for number of moves with English abilities as the only covariate.**

|  | χ^2^ | *df* |
| --- | --- | --- |
| CELF Core | 1.18 | 1 |
| Group | 0.14 | 1 |
| Condition | 13.24** | 2 |
| Phase | 0.43 | 1 |
| Group x Condition | 0.14 | 2 |
| Group x Phase | 0.34 | 1 |

**Significance level < 0.01

**Table 1b: Full regression model for number of moves with NST as reference group and English abilities as the only covariate.**

|  | *b* | *SE* | *t* |
| --- | --- | --- | --- |
| Intercept | 4.65 | 0.13 | 36.82* |
| CELF Core | -0.10 | 0.09 | -1.09 |
| Group | -0.01 | 0.26 | -0.04 |
| Condition-AST | 0.66 | 0.21 | 3.13* |
| Condition-MST | 0.56 | 0.18 | 3.09* |
| Phase | 0.10 | 0.15 | 0.65 |
| Group x Condition-AST | 0.11 | 0.43 | 0.26 |
| Group x Condition-MST | 0.13 | 0.36 | 0.36 |
| Group x Phase | 0.18 | 0.30 | 0.58 |

*Significance level < 0.05

**Table 2a: Wald chi-squared test of model improvement for number of moves with SES and English abilities as covariates.**

|  | χ^2^ | *df* |
| --- | --- | --- |
| CELF Core | 1.08 | 1 |
| SES | 0.22 | 1 |
| Group | 0.09 | 1 |
| Condition | 13.26** | 2 |
| Phase | 0.44 | 1 |
| Group x Condition | 0.14 | 2 |
| Group x Phase | 0.34 | 1 |

**Significance level < 0.01

**Table 2b: Full regression model for number of moves with NST as reference group and SES and English abilities as covariates.**

|  | *b* | *SE* | *t* |
| --- | --- | --- | --- |
| Intercept | 4.65 | 0.13 | 36.79* |
| CELF Core | -0.10 | 0.09 | -1.04 |
| SES | -0.04 | 0.09 | -0.47 |
| Group | 0.03 | 0.27 | -0.10 |
| Condition-AST | 0.67 | 0.21 | 3.14* |
| Condition-MST | 0.56 | 0.18 | 3.08* |
| Phase | 0.10 | 0.15 | 0.65 |
| Group x Condition-AST | 0.11 | 0.42 | 0.26 |
| Group x Condition-MST | 0.13 | 0.36 | 0.36 |
| Group x Phase | 0.18 | 0.30 | 0.59 |

*Significance level < 0.05

**Table 3a: Wald chi-squared test of model improvement for planning time with English abilities as the only covariate.**

|  | χ^2^ | *df* |
| --- | --- | --- |
| CELF Core | 0.79 | 1 |
| Group | 4.27* | 1 |
| Condition | 26.76*** | 2 |
| Phase | 42.13*** | 1 |
| Group x Condition | 6.02* | 2 |
| Group x Phase | 3.97* | 1 |

*Significance level < 0.05

***Significance level < 0.001

**Table 3b: Full regression model for planning time with NST as reference group and English abilities as the only covariate.**

|  | *b* | *SE* | *t* |
| --- | --- | --- | --- |
| Intercept | 4.77 | 0.19 | 24.99* |
| CELF Core | 0.12 | 0.14 | 0.89 |
| Group | -1.10 | 0.40 | -2.75* |
| Condition-AST | -0.50 | 0.20 | -2.53* |
| Condition-MST | 0.33 | 0.24 | 1.37 |
| Phase | -0.91 | 0.14 | -6.52* |
| Group x Condition-AST | 0.70 | 0.40 | 1.76 |
| Group x Condition-MST | 0.03 | 0.49 | 0.07 |
| Group x Phase | 0.55 | 0.29 | 1.99* |

*Significance level < 0.05

**Table 4a: Wald chi-squared test of model improvement for planning time with SES and English abilities as covariates.**

|  | χ^2^ | *df* |
| --- | --- | --- |
| CELF Core | 0.72 | 1 |
| SES | 0.16 | 1 |
| Group | 3.83 | 1 |
| Condition | 26.82*** | 2 |
| Phase | 42.14*** | 1 |
| Group x Condition | 6.03* | 2 |
| Group x Phase | 3.96* | 1 |

*Significance level < 0.05

***Significance level < 0.001

**Table 4b: Full regression model for planning time with NST as reference group and SES and English abilities as covariates.**

|  | *b* | *SE* | *t* |
| --- | --- | --- | --- |
| Intercept | 4.77 | 0.19 | 24.88* |
| CELF Core | 0.12 | 0.14 | 0.85 |
| SES | 0.05 | 0.13 | 0.40 |
| Group | -1.08 | 0.40 | -2.68* |
| Condition-AST | -0.50 | 0.20 | -2.53* |
| Condition-MST | 0.33 | 0.24 | 1.37 |
| Phase | -0.91 | 0.14 | -6.52* |
| Group x Condition-AST | 0.70 | 0.40 | 1.76 |
| Group x Condition-MST | 0.03 | 0.49 | 0.07 |
| Group x Phase | 0.55 | 0.28 | 1.99* |

*Significance level < 0.05

**Table 5a: Wald chi-squared test of model improvement for execution time with English abilities as the only covariate.**

|  | χ^2^ | *df* |
| --- | --- | --- |
| CELF Core | 3.12 | 1 |
| Group | 0.09 | 1 |
| Condition | 67.79*** | 2 |
| Phase | 0.20 | 1 |
| Group x Condition | 1.14 | 2 |
| Group x Phase | 0.50 | 1 |

*Note.* For model convergence, the random by-subject slope of learning was removed.

***Significance level < 0.001

**Table 5b: Full regression model for execution time with NST as reference group and English abilities as the only covariate.**

|  | *b* | *SE* | *t* |
| --- | --- | --- | --- |
| Intercept | 9.18 | 0.46 | 20.10* |
| CELF Core | -0.63 | 0.35 | -1.77 |
| Group | -0.51 | 0.96 | -0.54 |
| Condition-AST | 2.44 | 0.66 | 3.67* |
| Condition-MST | 5.40 | 0.66 | 8.19* |
| Phase | -0.24 | 0.51 | -0.46 |
| Group x Condition-AST | -0.11 | 1.33 | -0.08 |
| Group x Condition-MST | 1.26 | 1.32 | 0.96 |
| Group x Phase | 0.72 | 1.02 | 0.71 |

*Note.* For model convergence, the random by-subject slope of learning was removed.

*Significance level < 0.05

**Table 6a: Wald chi-squared test of model improvement for execution time with SES and English abilities as covariates.**

|  | χ^2^ | *df* |
| --- | --- | --- |
| CELF Core | 2.95 | 1 |
| SES | 0.13 | 1 |
| Group | 0.15 | 1 |
| Condition | 67.90*** | 2 |
| Phase | 0.19 | 1 |
| Group x Condition | 1.14 | 2 |
| Group x Phase | 0.47 | 1 |

***Significance level < 0.001

**Table 6b: Full regression model for execution time with NST as reference group and SES and English abilities as covariates.**

|  | *b* | *SE* | *t* |
| --- | --- | --- | --- |
| Intercept | 9.18 | 0.46 | 19.96* |
| CELF Core | -0.61 | 0.36 | -1.72 |
| SES | -0.12 | 0.33 | -0.35 |
| Group | -0.55 | 0.97 | -0.57 |
| Condition-AST | 2.43 | 0.66 | 3.68* |
| Condition-MST | 5.40 | 0.66 | 8.20* |
| Phase | -0.23 | 0.53 | -0.44 |
| Group x Condition-AST | -0.11 | 1.32 | -0.08 |
| Group x Condition-MST | 1.26 | 1.32 | 0.96 |
| Group x Phase | 0.72 | 1.05 | 0.69 |

*Significance level < 0.05

**Table 7a: Wald chi-squared test of model improvement for accuracy on secondary task with English abilities as the only covariate.**

|  | χ^2^ | *df* |
| --- | --- | --- |
| CELF Core | 4.05* | 1 |
| Group | 1.48 | 1 |
| Condition | 110.19*** | 1 |
| Group x Condition | 0.12 | 1 |

*Significance level < 0.05

***Significance level < 0.001

**Table 7b: Full regression model for accuracy on secondary task with English abilities as the only covariate.**

|  | *b* | *SE* | *t* |
| --- | --- | --- | --- |
| Intercept | 0.79 | 0.02 | 54.15* |
| CELF Core | 0.02 | 0.01 | 2.01* |
| Condition | 0.27 | 0.03 | 10.49* |
| Group | 0.20 | 0.03 | 0.67 |
| Group x Condition | 0.02 | 0.05 | 0.35 |

*Significance level < 0.05

**Table 8a: Wald chi-squared test of model improvement for accuracy on secondary task with SES and English abilities as covariates.**

|  | χ^2^ | *df* |
| --- | --- | --- |
| CELF Core | 2.77 | 1 |
| SES | 0.75 | 1 |
| Group | 2.37 | 1 |
| Condition | 103.09*** | 1 |
| Group x Condition | 0.06 | 1 |

***Significance level < 0.001

**Table 8b: Full regression model for accuracy on secondary task with SES and English abilities as covariates.**

|  | *b* | *SE* | *t* |
| --- | --- | --- | --- |
| Intercept | 0.78 | 0.01 | 52.77* |
| CELF Core | 0.02 | 0.01 | 1.66 |
| SES | 0.01 | 0.01 | 0.86 |
| Condition | 0.27 | 0.03 | 10.14* |
| Group | 0.03 | 0.03 | 1.01 |
| Group x Condition | 0.01 | 0.05 | 0.25 |

*Significance level < 0.05
